# Liquid Metal-Based Devices: Material Properties, Fabrication and Functionalities

**DOI:** 10.3390/nano11123400

**Published:** 2021-12-15

**Authors:** Jian Dong, Yuanyuan Zhu, Zhifu Liu, Meng Wang

**Affiliations:** School of Computer Science and Engineering, Central South University, Changsha 410075, China; dongjian@csu.edu.cn (J.D.); zyyhhh@csu.edu.cn (Y.Z.); 194712106@csu.edu.cn (Z.L.)

**Keywords:** liquid metal, flexible circuits, reconfigurable antenna, wearable devices, strain sensors, 3-D printing, spray printing, microfluidic channel

## Abstract

This paper reviews the material properties, fabrication and functionalities of liquid metal-based devices. In modern wireless communication technology, adaptability and versatility have become attractive features of any communication device. Compared with traditional conductors such as copper, the flow characteristics and lack of elastic limit of conductive fluids make them ideal alternatives for applications such as flexible circuits, soft electronic devices, wearable stretch sensors, and reconfigurable antennas. These fluid properties also allow for innovative manufacturing techniques such as 3-D printing, injecting or spraying conductive fluids on rigid/flexible substrates. Compared with traditional high-frequency switching methods, liquid metal (LM) can easily use micropumps or an electrochemically controlled capillary method to achieve reconfigurability of the device. The movement of LM over a large physical dimension enhances the reconfigurable state of the antenna, without depending on nonlinear materials or mechanisms. When LM is applied to wearable devices and sensors such as electronic skins (e-skins) and strain sensors, it consistently exhibits mechanical fatigue resistance and can maintain good electrical stability under a certain degree of stretching. When LM is used in microwave devices and paired with elastic linings such as polydimethylsiloxane (PDMS), the shape and size of the devices can be changed according to actual needs to meet the requirements of flexibility and a multistate frequency band. In this work, we discuss the material properties, fabrication and functionalities of LM.

## 1. Introduction

Due to the high thermal conductivity, low viscosity, and high fluidity of liquid metal, LM has become an ideal candidate for various fields, such as flexible electronics, thermal management, soft machines, and biomedical materials. LM has been integrated with micro/nano technology, so that LM has significantly diversified properties. These innovative functional materials not only have the softness of classic liquid metals, but also have many outstanding properties, including self-healing ability and stimulus-response deformation ability. Compared with rigid inorganic micro/nano-level materials, soft liquid metal micro/nano-particles exhibit exceptional superior flexibility, compliance and adjustability [1].

The fluid properties of LM allow for innovative manufacturing techniques such as 3-D printing. An LM array based on 3-D printing was described in [2]. The patch antenna array and the insulated part of the integrated feed network were printed using a ProJet 3500HDMax MultiJet printer. The printer supports coprinting of VisiJet M3 Crystal (3-D Systems) acrylic resin and VisiJet S300 (3-D Systems) support material by immersing the printed part in a warm ultrasonic cleaning tank (EZRinse-C, 3-D Systems) maintained at 55 °C, dissolving and flushing the sacrificial support through the small drainage hole. The prototype was kept in a vacuum oven at 55 °C for about 12 h to remove the solvent, and then the drain hole was sealed by applying photocurable polymer NOA63 (Norland Optical Adhesive 63). The cavity in the acrylic structure was filled with eutectic gallium indium (EGaIn), and a vacuum-driven process was used to form conductive elements. This process was a convenient method to rapidly prototype the complex array design to improve its electromagnetic performance. In addition to 3-D printing technology, the LM microfluidic channel also uses soft lithography technology. In the EGaIn thin line pattern production method based on soft lithography technology, the size of the produced thin lines can be scalable, uniform and without residue, and the line width on the same soft substrate can be from a single micron to a few millimeters [3]. Spray printing can be directly applied to various substrates, and liquid metal droplets can be sprayed on the cloth substrate [4], sprayed and wiped on the PDMS substrate [5], or quantitatively sprayed and packaged in silicone elastomer in combination with silicone [6].

When LM is used as the switching element of the antenna, different positions of the liquid metal in the antenna structure represent the on or off state, thereby connecting or disconnecting different structures in the antenna, changing the current distribution on the antenna to achieve different antenna performance. In [7], a broadband reconfigurable cone antenna using an LM reflector was proposed, and liquid metal was used as the switching mechanism to realize beam steering. When the channels in different positions were injected with LM as a reflector, the radiation of the cone antenna was steered to the opposite direction of the channels to achieve beam steering. The injector was used to control the motion of the LM effectively, and a wide bandwidth from 1.7 to 2.7 GHz and a stable radiation pattern that could perform 21 beam steering under 360° coverage were obtained. This had a simple design, wide impedance bandwidth, and a maximum measurement gain of 6.7 dB, which can be used in LTE applications and indoor rooftop antenna network applications. Using liquid metals to connect/disconnect large-area metalization can achieve radiation performance that is impossible with traditional switches.

In liquid metal-based reconfigurable antennas, alterations in the length and position of the LM in a large amplitude greatly enhance the reconfigurable state of the devices, and with an extensive frequency adjustment range. The realization of these reconfigurable states does not depend on nonlinear materials or mechanisms, compared to antennas using semiconductor switches, and these antennas also have higher linearity. A hybrid frequency and polarization reconfigurable slot antenna using LM was proposed [8]. A PDMS structure with narrowband microchannels was loaded on the printed circuit board (PCB) of the square slot antenna to provide conditions for reconstruction. Left-hand circular polarization (LHCP), right-hand circular polarization (RHCP) and linear polarization (LP) were achieved by injecting +45° and −45° channels relative to the x-axis or keeping the channels empty. By filling microchannels of different lengths, three frequency reconfigurations in LHCP and RHCP states could be realized. The antenna also had the advantages of wide bandwidth and small size of a circularly polarized antenna which can be applied to other potential topologies with various reconfigurable functions.

There is growing interest in using LMs as tuning components for radio frequency/microwave applications, such as filters [9,10,11,12], resonators [13] and frequency selective surfaces (FSS) [14,15]. Strain sensors are essential to study the changes of the human body in the medical field. A large part of human body sign parameters is the generation of stress or strain, such as heart rate, facial expression, joint movement of fingers and knees [16,17]. Therefore, when measuring these physical parameters, people usually measure the frequency or magnitude of the stress or strain at the corresponding position, thus stress-strain sensors are a critical category of LM sensors. The development of science and technology has brought a huge demand for flexible electronic devices such as electronic skins, wearable devices, and soft robots [18]. LM has gradually entered people’s view as a flexible conductive material and has become a research hotspot. 

This review focuses on the aspects of LM, including material properties, fabrication, and functionalities. In Section 2 of this work, the material properties of LM are presented. Later in Section 3, various fabrication methods are discussed. The functionalities of LM are summarized in Section 4. Finally, we discuss and conclude in Section 5.

## 2. Material Properties

LM is a metal or alloy that has a relatively low melting point compared to traditional solid metals and can remain in the liquid state at room temperature (melting point < 30 °C). Room temperature liquid metals mainly include mercury (Hg, melting point −38.8 °C), gallium (Ga, ~29.8 °C), rubidium (Rb, ~38.9 °C), francium (Fr, ~27 °C), and cesium (Cs, ~28.4 °C). Among them, mercury is the most common LM in daily life. Although it has good adhesion properties, it is easy to evaporate produces highly toxic mercury vapor. Rubidium, francium and cesium are highly radioactive and chemically unstable, which makes their applications greatly restricted. For example, cesium can explode violently with hydrogen [19]. Gallium with a low melting point (29.8 °C) and high boiling point (2204 °C) is relatively stable and has been widely used and studied.

The liquid metals discussed in this paper are mainly gallium-based liquid metal alloys. Eutectic gallium indium (75.5% Ga, 24.5% In) and Galinstan (68.5% Ga, 21.5% In, 10% Sn) have lower melting points than elemental metal gallium. Different ratios of the alloy affect the melting point and other physical properties of LMs, as shown in Table 1. The preparation of gallium-based LM is quite simple. Here, EGaIn composed of 75.5 wt% gallium and 24.5 wt% indium is taken as an example, and its melting point is 15.5 °C. Firstly, preweighed gallium (75.5 g) is put into a beaker and heated in a water bath (60 °C) for 10 min so that the gallium is melted. Then, indium (24.5 g) is added to the beaker, and the Ga-In mixture is continuously stirred with a magnetic stirrer at 80 °C for 1 h until uniformly mixed [20]. Commercially available Galinstan, composed of 68.5% gallium, 21.5% indium and 10% tin, is also a commonly used liquid metal alloy, with a lower melting point than room temperature (−19 °C) and nontoxic. When the gallium-based alloy is exposed to the air, the gallium metal rapidly oxidizes, and the formed oxide layer is located on the surface of the metal droplet, enveloping the entire metal droplet. On the one hand, it avoids further oxidation of the metal inside, and on the other hand, it can maintain the integrity of the droplets. This feature makes the process of applying LM more convenient. In addition, as the most biocompatible category of all LMs, gallium-based LM has a physical and chemical properties such as fluidity, electrical conductivity and thermal conductivity that are not inferior to other LMs, which makes gallium-based LM the most suitable type of liquid metal to be promoted [21].

It should be noted that although LM fluid has the main flexibility advantages, including the long-term stability of its electrical properties, it is necessary to consider the corrosiveness of Ga to almost all metals (except tungsten (W) and tantalum (Ta)) before using EGaIn and Galinstan in devices. This corrosivity can be overcome by modern composite materials such as a graphene oxide (GO) layer with poly 3, 4-ethyldioxythiophene: polystyrene sulfonate (PEDOT: PSS). In the composite material PEDOT: PSS/GO, graphene oxide prevents Ga from contacting the metal surface, while PEDOT: PSS overcomes the nominal insulating properties of graphene oxide and ensures good electrical contact between the metal interconnection pin and the LM alloy [22]. The surface of pure Ga or eutectic Ga-based alloy is easily oxidized and forms an amorphous Ga oxide layer in the environment, thereby reducing the surface tension of the liquid metal. The thickness of the Ga oxide surface layer formed is 0.5–3 nm, which can be adjusted by using an electrochemical method. The surface oxide layer can be eliminated by applying an electrochemical reduction potential, or simply by a diluted acid/strong alkaline medium [23].

## 3. Fabrication

### 3.1. 3-D Printing

3-D printing is a kind of rapid prototyping technology, also known as additive manufacturing. It is a technology that builds objects based on digital model files and prints them layer by layer with appropriate materials. There are many different technologies for 3-D printing, such as Fused Deposition Modeling (FDM), Binder Jet (BJ), Selective Laser Sintering (SLS), Selective Laser Melting (SLM) and other technologies [24], and a variety of materials are used, such as metals, polymers, and composite materials.

**Figure 1:**
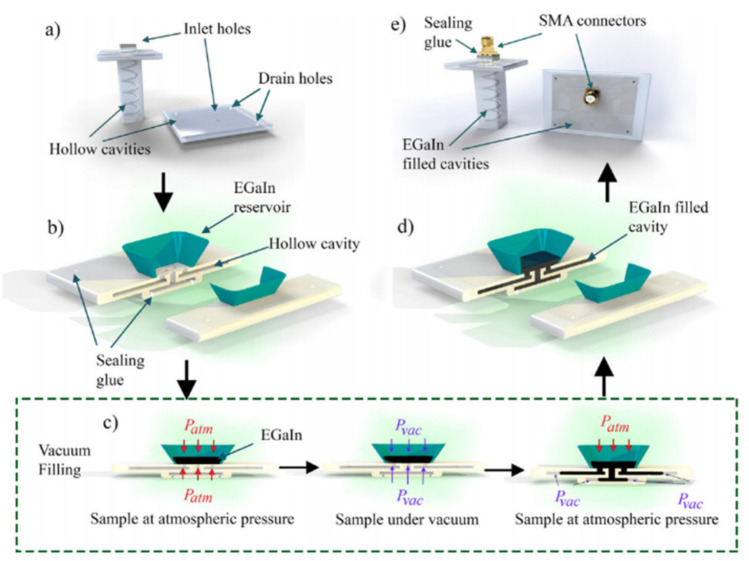
Manufacturing process diagram of liquid metal-filled 3-D printed antenna. (**a**) 3-D printed patch and helix antenna structures with sacrificial wax removed. (**b**) Snap-off liquid storage tank for filling liquid metal and sealing drainage holes. (**c**) The patch antenna is filled with liquid metal under vacuum, and the metal is pushed into the channel by the pressure differential. (**d**) A patch antenna filled with liquid metal. (**e**) Sealing the SMA connectors to finish the antenna manufacturing process. Reprinted with permission from Ref. [25]. Copyright 2017 Elsevier.

LM is usually combined with 3-D printing technology. The common method is to fill the 3-D printed channels/cavities with LM. Three-dimensional printing is an additive process that prints the complex shell structure (microfluidic channel) of the antenna on the target substrate, mainly using the FDM method. In [25], in order to manufacture a complex three-dimensional antenna, as shown in Figure 1, a cavity was made by 3-D printing, and a wax-like support material was used to ensure the independent geometric shape of the area to be metalized. After printing the cooled parts, an ultrasonic cleaning tank was used with cleaning fluid to dissolve the printing wax holder at about 55 °C. The dissolved cavity was placed in a vacuum, and the liquid metal was pushed into the cavity by pressure difference. 

In [26], a wearable strain sensor was designed in which a gallium-based liquid metal paste was 3-D printed to obtain better stability and facilitate extrusion flow. To change the performance, nickel nanoparticles and an ultrasonic treatment were added. In [27], a two-dimensional tunable frequency selective absorber containing LM and sodium hydroxide solution was proposed. The structure consisted of a circular cavity and a three-dimensional printed substrate. It contained small steps, in which the radius of the EGaIn could be controlled by electrochemical methods. Ref. [28] proposed a new method for manufacturing microwave devices using LM conductors and three-dimensional printed dielectric containers, and the 3-D printed conductive media materials provided electrical connections with LM at input and output ports.

**Figure 2:**
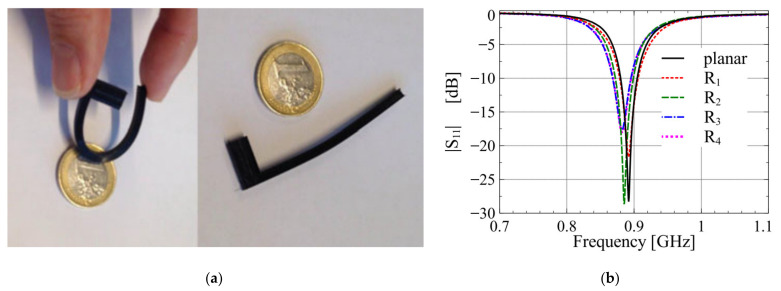
(**a**) NinjaFlex 3-D printed sample in bent and relaxed state. (**b**) Measured impedance matching for different radii of curvature. Reprinted with permission from Ref. [29]. Copyright 2017 IEEE.

A miniaturized inverted F antenna (IFA) was designed, implemented and measured in [29]. During the manufacturing process, Fused Deposition Modeling (FDM) technology was used to manufacture the NinjaFlex flexible plastic dielectric radome, and the microchannels were filled with LM. Galinstan was selected for the LM, and the NinjaFlex flexible plastic was printed through a 3-D FDM process to achieve a dielectric substrate encapsulating the LM. The antenna worked at 885 MHz and had low sensitivity to bending. As shown in Figure 2b, the resonant frequency was quite stable, and the maximum frequency shift was about 1%.

**Figure 3:**
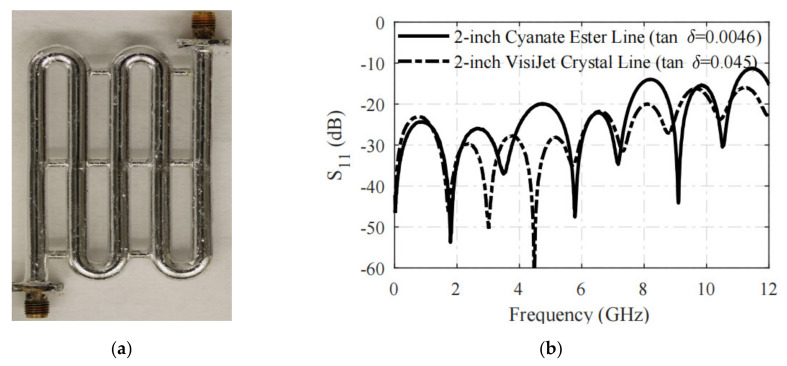
(**a**) A 3-D printed 2-ns delay line. (**b**) Measured S_11_ result of 2-inch 3-D printed coaxial lines using different dielectric material. Reprinted with permission from Ref. [30]. Copyright 2018 IEEE.

When using 3-D printing technology to manufacture coaxial transmission lines, ref. [30] introduced a new method. As reported in Figure 3a, the dielectric part was 3-D printed with low loss factor resin cyanate ester (tanδ = 0.0046). The inner conductor was formed by pumping EGaIn through the empty channel of the dielectric part, and the outer conductor was electrophoretically plated with silver and then coated with EGaIn. After manufacturing and measuring, it was found that the low loss factor dielectric material could achieve low loss performance. As shown in Figure 3b, the cyanate ester wire had a lower reflection loss than the VisiJet crystal M3 dielectric. The use of EGaIn provided a high degree of design freedom for coaxial structures. In future work, reconfigurable coaxial design will be considered to take advantage of the benefits of EGaIn.

### 3.2. Soft Lithography

In addition to 3-D printing technology, the LM microfluidic channel uses soft lithography technology. The core of soft lithography is to make an elastic stamp, which can be obtained quickly and efficiently through photolithography and molding methods and uses an elastic mold to produce microstructures, micro molds and micro fluids. The most commonly used elastic mold stamp material for soft lithography is PDMS. Compared with traditional lithography technology, soft lithography technology has greater flexibility because it can make multi-layer structure or three-dimensional structure and can also make molds on irregular surfaces.

**Figure 4:**
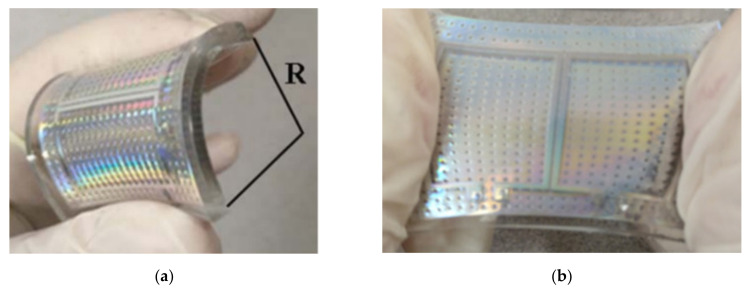

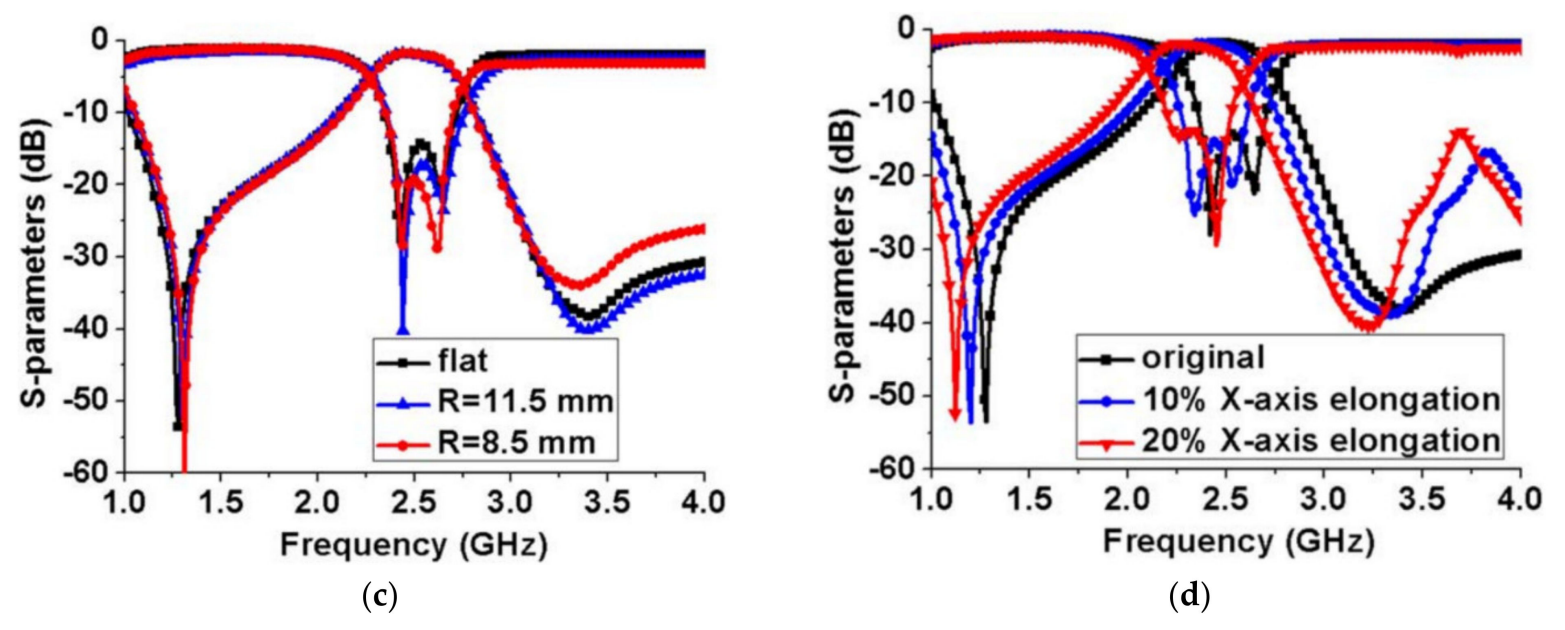
(**a**) Fabrication process of the bandpass filter. (**b**) Photograph of the bended filter. (**c**) Measured S-parameters of the flat filter and the bended filters. (**d**) Measured S-parameters of the original and the stretched filters. Reprinted with permission from Ref. [31]. Copyright 2018 John Wiley and Sons.

A flexible LM alloy bandpass filter (BPF) was described in Figure 4 [31]. The filter used soft lithography technology with multilayer lamination capabilities. Galinstan was selected as the LM and injected into the microfluidic channel through a syringe to form a complete filter. Figure 4c shows different situations with different curvature radii (R) in Figure 4a. In the measurement results of the plane and the curved filter with R = 8.5 mm and 11.5 mm, it can be seen that the curvature of the filter had little effect on the resonance frequency. When the filter was stretched from the original state to 10–20%, as shown in Figure 4b, its center frequency moved from 2.48 GHz to 2.42–2.36 GHz in Figure 4d. This showed that in the case of stretching, the increase in the size of the resonator of the filter results in a decrease in the resonant frequency. Experiments have studied the flexibility of the filter, including bending, twisting and stretching capabilities. The filter was not sensitive to bending and twisting, and the stretching behavior had a slight influence on its center frequency.

A pressure sensor based on LM was proposed in [32]. The sensor consisted of three parts: spring steel shell (50CrVA), silicone rubber, and PDMS elastomer, in which EGaIn filled microchannels produced by laser soft lithography were embedded. When pressure was applied to the surface of the pressure sensor, the deformation of the microchannel caused a change in resistance and established the relationship between resistance R and pressure P, effectively increasing the working range of the flexible pressure sensor.

**Figure 5:**
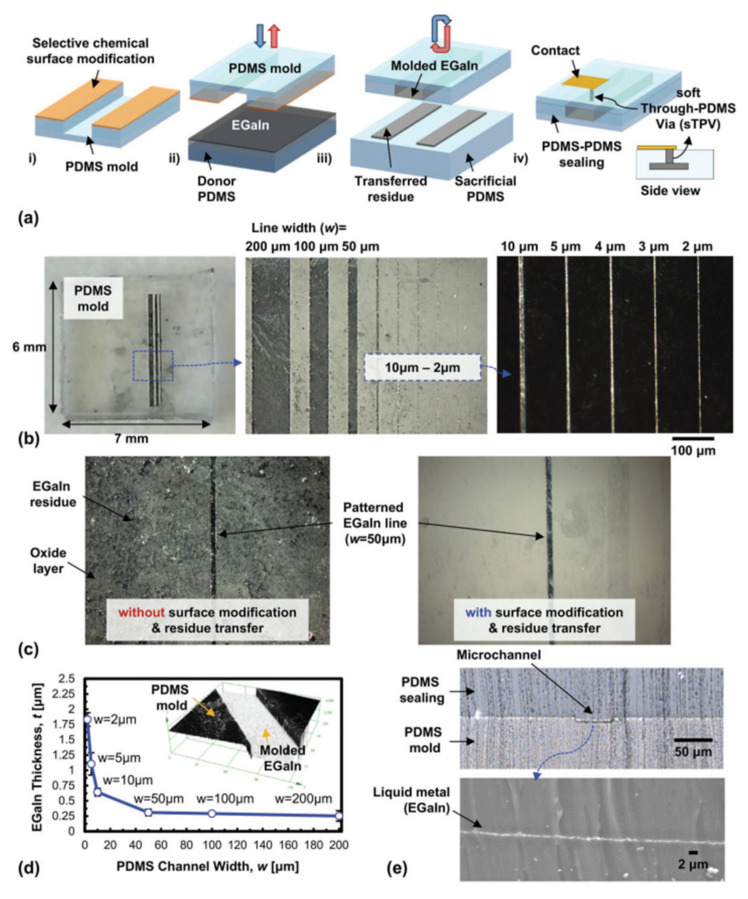
(**a**) Patterning process of EGaIn thin-line fabrication. (**b**) The widths of EGaIn lines from 2 µm to 200 µm with channel spacings of 100 µm. (**c**) The Patterned EGaIn line and surrounding PDMS surfaces with and without chemical surface modification and residue transfer technique for residue-free surfaces. (**d**) Measured EGaIn thickness inside of a PDMS mold as a function of PDMS channel width. (**e**) Cross-sectional view of EGaIn molded microchannel (top) and enlarged view of EGaIn molded thin film (bottom). Reprinted with permission from Ref. [3]. Copyright 2016 John Wiley and Sons.

A method of making EGaIn thin line patterns was proposed in [3]. The method was based on soft lithography technology, and the thin-line size was scalable, uniform and residue-free, allowing the line width on the same soft substrate to range from single micrometers to a few millimeters. The LM thin line pattern process made it possible to expand and contract soft microelectronic components and circuits and construct more complex and flexible hybrid electronic devices. The soft lithography process based on LM included four steps. As shown in Figure 5, the PDMS mold was selectively chemically modified to increase the hydrophobicity of the surface, and any unwanted LM residue outside the channel area was removed. Finally, a PDMS mold that filled the channel with LM was covered with an additional PDMS layer.

### 3.3. Spray Printing

Printed electronics technology based on printing principles has the advantages of fastness, flexibility, individual customization, and low cost. At present, there are three typical conductive inks in printed electronic products: conductive carbon ink, conductive polymer, and metal conductive ink. However, the conductivity of conductive carbon ink and conductive polymer is generally lower than 10^5^ S/m, which is not suitable for application in the manufacture of high-sensitivity circuits. For metal conductive inks, a long-term curing process is required for post processing, which reduces the printing speed and increases the manufacturing cost. As an alternative, the LM conductive ink is becoming an important research focus [33].

**Figure 6:**
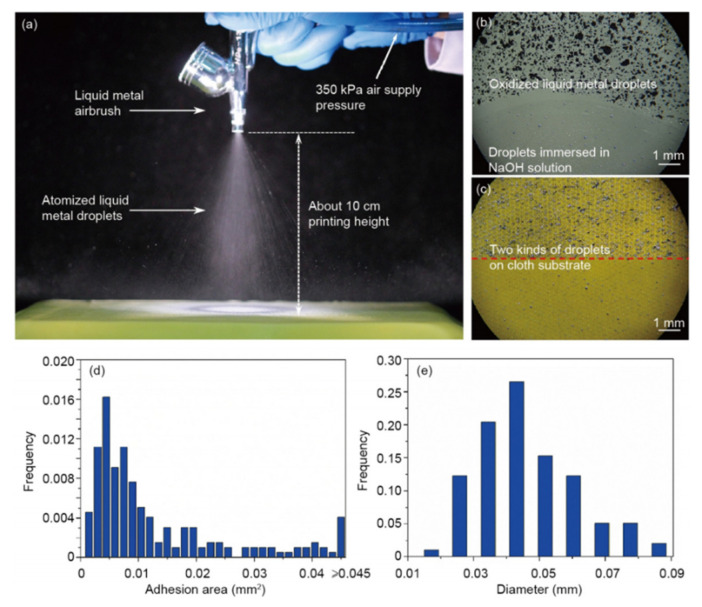
(**a**) Droplets generated by the airbrush. Light microscope image of spraying oxidized LM droplets and reduced droplets by immersing in NaOH solution on transparent glass substrate (**b**) and on cloth substrate (**c**). Statistical graph of the size distribution of oxidized droplets after impacting the substrate (**d**) and the droplets after removing their oxidized layer and shrinking into spheres (**e**). Reprinted with permission from Ref. [4]. Copyright 2016 Springer Nature.

Spray printing can be directly applied to various substrates, and atomized LM can be printed on almost any desired substrate. The LM in the spray brush is decomposed by high-pressure nitrogen into droplets [4]. As shown in Figure 6, these droplets were mixed with nitrogen and exposed to the air to form a thin oxide film on the surface. When these oxidized droplets hit the substrate, good adhesion and special morphological structures were observed. After being sprayed on the substrate, the LM droplets showed an irregular shape with rotating edges. A small amount of 0.5 mol/L NaOH can be dropped gently on the substrate. The metal droplets immersed in the solution restore the original luster and shrink into a spherical shape in situ due to the elimination of the surface oxide film. Using the above steps, LM droplets can be sprayed on the cloth substrate and the oxide film can be removed by immersing in a NaOH solution. LM spray printing is of great significance in the development of wearable electronic textiles.

A new method of manufacturing liquid-metal circuits on PDMS by spraying and wiping processes proposed in [5] was designed with a special microchannel thar could be used to prepare a PDMS substrate in advance to make a LM entity formed by subsequent spraying and wiping. Experiments showed that the whole process was easy to implement, convenient to operate, and simultaneously provided a simpler manufacturing process and smaller feature sizes. Although spraying technology has been used to deposit many materials, Ref. [6] reported a spray deposition technology that allowed LM alloys to form stretchable conductors that were encapsulated in silicone elastomers. The focus of this work was the quantitative spraying process and combining it with silicone.

## 4. Functionalities

As a new type of alloy material, LM not only has high conductivity, but also has the natural advantages of good fluidity and controllable deformation that traditional solid metals do not have. Therefore, LM has been widely used and has become a research hotspot at home and abroad in recent years.

### 4.1. Liquid Metal Switches

So far, many switching technologies have been proposed, including mechanical switches [34], radio frequency micro-electromechanical systems (MEMS) technologies [35], water-based absorption switches [36] and magnetic locking switches [37]. When LM is used as the switching element of the antenna, different positions of the LM in the antenna structure represent the on or off state, thereby connecting or disconnecting different structures in the antenna, and changing the current distribution on the antenna to achieve different antenna performance. Due to its excellent radio frequency performance and linear behavior, LM has been used for switches.

**Figure 7:**
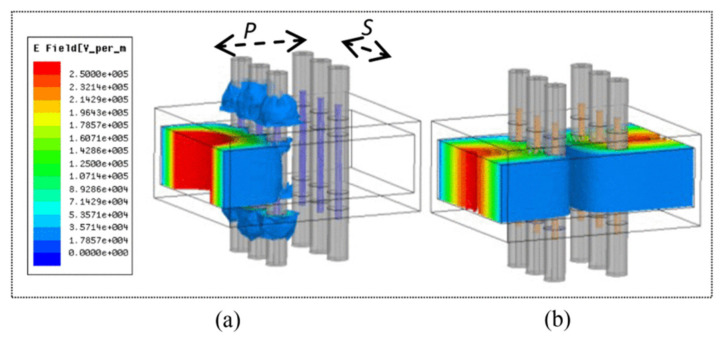
Electric field distribution of the proposed microfluidic switch: (**a**) OFF state (LM inserted); (**b**) ON state (LM removed). Reprinted with permission from Ref. [38]. Copyright 2017 IEEE.

Ref. [38] introduced a k-band reflective waveguide switch using EGaIn. The microfluidic channel connected to the micropump was inserted into a WR-42 waveguide along the wide wall. The microfluidic channel carried the LM suspended in the oil carrier. By inserting/removing the metal post in the wide wall of the waveguide, as shown in Figure 7, the switch was closed and opened. When the switch was open, the metal pillar was located outside the waveguide, allowing almost undisturbed wave propagation; when the switch was closed, the LM pillar was pumped into the waveguide and created a virtual conductive wall that blocked signal propagation. Under the operating frequency of 20 GHz, the insertion loss was as low as 0.1 dB (ON state) and the isolation loss was greater than 30 dB (OFF state). Compared with mechanical switches widely used in millimeter wave communications, this method provided a low-cost and compact alternative.

Likewise, gallium-based LM can be used as a substrate integrated waveguide switch, which was used in the application of reconfigurable microwave devices [39]. The propagation of waves was allowed or prohibited by using movable walls constructed from a series of boreholes. When walls were needed, the holes were filled with gallium-based LM. When the wall was no longer needed, these holes would empty the LM. This method was applied to single-pole single-throw (SPST) SIW switches and single-pole double-throw (SPDT) switches. The SPST switch worked from 2.4 GHz to 4.3 GHz. In the open state, the insertion loss (IL) was ≤0.5 dB, and in the closed state the isolation value was 30 dB. The SPDT switch operated from 4.7 GHz to 7.2 GHz, and the IL between the two connection ports was 0.7 dB. The isolation distance between unconnected ports was 40 dB. In terms of bandwidth, IL and isolation, the performance of the proposed switches was competitive and suitable for applications requiring high power handling capability and low loss. 

Different from the insertion and removal of LM in the aforementioned waveguide switch, a LM switch driven by continuous electrowetting was designed and simulated in [40]. Continuous electrowetting (CEW) refers to a section of LM immersed in the electrolyte under a certain applied voltage in which the metal-electrolyte surface produces a tension gradient that leads to the flow of this section of LM in the electrolyte. In [41], a LM radio frequency shunt switch was proposed. The switch combined a dielectric spacer on the radio frequency path with a capillary groove filled with electrolyte, in which the dielectric spacer can reduce insertion loss and realize low-power electric drive. The switch can achieve high isolation of 20 to 30 dB from DC to 5 GHz, and the isolation from 5 to 11 GHz was greater than 10 dB. The switch had a low insertion loss of 0.2 to 1.2 dB from DC to 5 GHz. Heat dissipation is a key obstacle to the realization of reliable, high-power-density electronic systems. Thermal devices that can actively manage heat transfer may optimize heat dissipation and improve reliability through thermalization of the device. LM has good thermal conductivity, as shown in Table 1, and hence [42] developed a millimeter-scale LM droplet thermal switch that can control heat transfer spatially and temporally. The thermal switch was integrated with a gallium nitride (GaN) device mounted on a printed circuit board (PCB), and the heat transfer and temperature of different switch positions and heat dissipation levels were measured. When integrated with a single GaN device (2.6 mm × 4.6 mm surface area), the thermal switch was 1.3 W at 51 °C ± 1 °C on mode. The GaN device at 95 °C ± 1 °C performed 0.5 W in the off mode to actively control the heat transfer capability. A one-dimensional system thermal resistance model was developed, combined with an independent three-dimensional finite element method (FEM) simulation, which was in good agreement with the experimental data.

**Figure 8:**
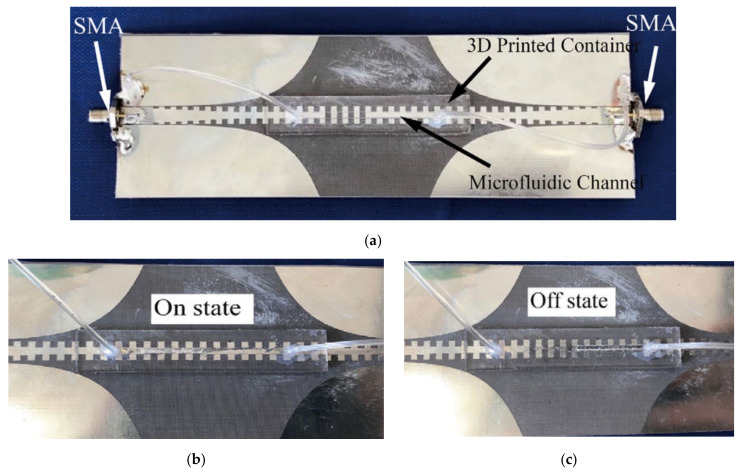

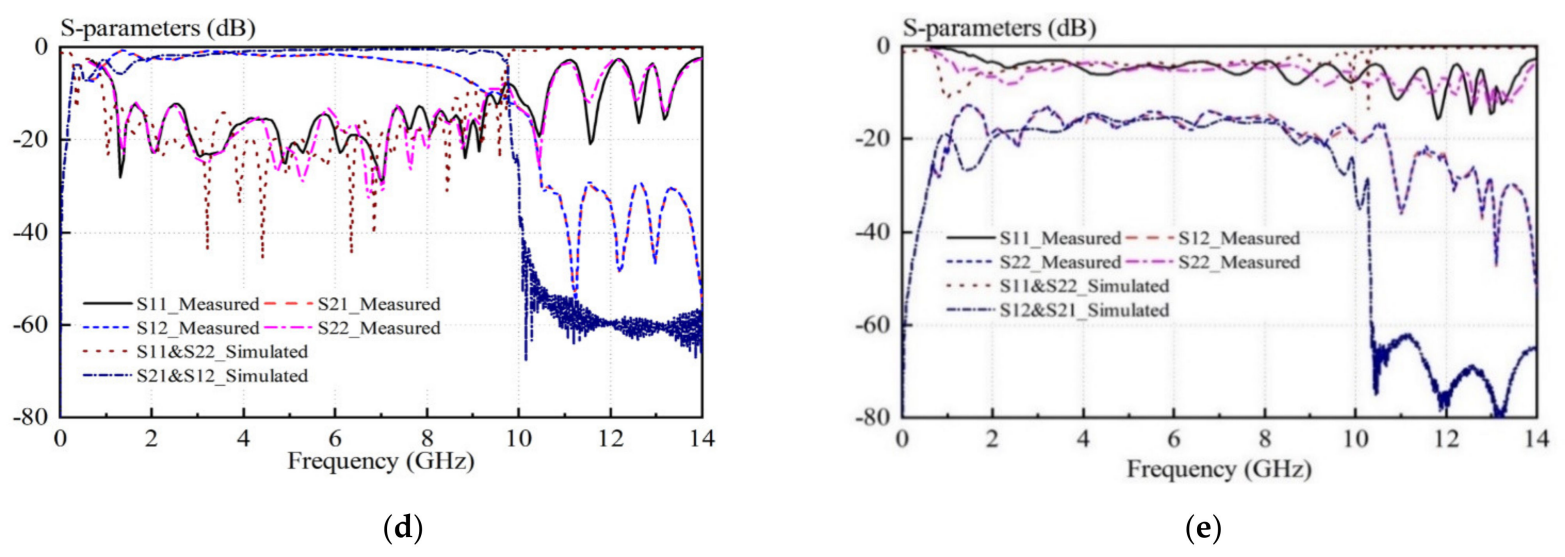
(**a**) Fabricated SPP transmission line. (**b**) SPP-based switch in ON-state by injecting LM to the channel and covering the cut unit cells. (**c**) SPP-based switch in OFF-state by injecting LM to the channel, which cannot cover the cut unit cells. (**d**) Simulated and measured S-parameters of the ON state. (**e**) Simulated and measured S-parameters of the OFF state. Reprinted with permission from Ref. [43]. Copyright 2021 John Wiley and Sons.

A radio frequency (RF) LM switch based on a spoof surface plasmon (SPP) transmission line is depicted in Figure 8 [43]. The LM in the syringe was separated from the air with mineral oil. After the LM was injected, its movement depended on the volume of the injected mineral oil. Using the good fluidity and high conductivity of LM, the signal was successfully blocked by cutting several units. The SPP transmission line can be modified by moving LM plug through microfluidic technology to obtain switches in different states. In the operating frequency band of 2–8 GHz, the insertion loss in the ON state was less than 1.5 dB and the isolation in the OFF state was greater than 13 dB. With the help of a higher precision manufacturing method and tuning the size of the proposed switch, its frequency band can be extended to millimeter wave band and even the terahertz band.

**Figure 9:**
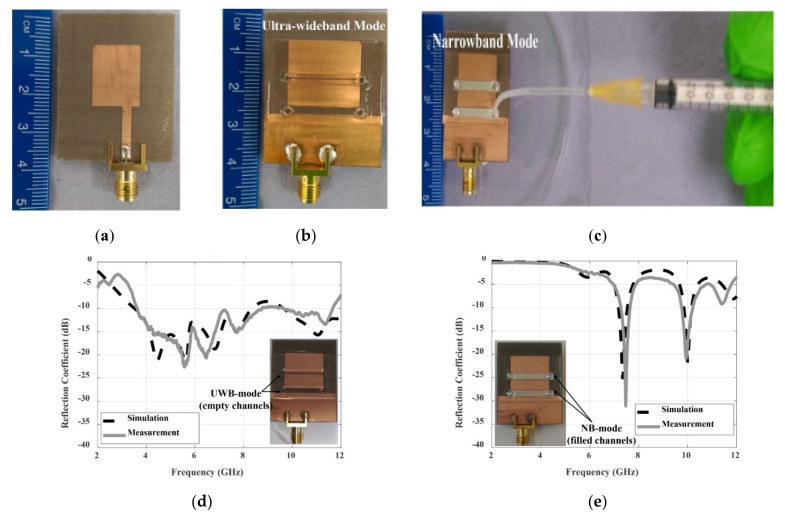
(**a**) Front view of the fabricated antenna. (**b**) Rear view of UWB mode. (**c**) Rear view of NB mode. (**d**) Reflection coefficient of UWB operating state. (**e**) Reflection coefficient of NB operating state. Reprinted with permission from Ref. [44]. Copyright 2020 IEEE.

In [44], the antenna can be switched between ultra-wideband (UWB) and narrowband (NB) by connecting/disconnecting the ground plane of the feeder line and the radiator feeder line, as depicted in Figure 9. Compared with the traditional semiconductor switch, the LM switch here can avoid the electrical contact gap between the traditional semiconductor switch, so that back radiation can be significantly reduced. The achieved gain and total efficiency were increased by 2 dBi and 24%, respectively. Therefore, the antenna had potential application prospects in wireless systems using cognitive radio (CR) and spectrum aggregation. 

### 4.2. Liquid Metal Reconfigurable Antennas

Mobile communication systems are developing towards multifrequency and multimode, with the size of mobile devices getting smaller and smaller. The development of mobile internet technology has created a large demand for small wireless systems resulting in a challenge to design an antenna that meets all requirements because the size of the antenna and the operating frequency band conflict with each other. The traditional multi-antenna system increases the occupied area and load weight of the communication platform. What is more serious is that the electromagnetic interference problem between the various antennas in the multi-antenna system deteriorates the working performance of the antennas. Compared with the multi-antenna system, the reconfigurable antenna can realize the function of the multi-antenna system on the same or even a smaller physical aperture, which meets the current development trend of multimode, multifrequency and increasingly miniaturized communication systems.

Traditional reconfigurable antennas mainly use mechanical changes in structure or materials with variable electromagnetic properties to change the current distribution of the antenna in different states, so as to achieve reconfiguration of frequency/radiation pattern and polarization [45]. Among them, electronic devices are the most commonly used reconfigurable components due to their fast speed of switching and easy integration into antennas. However, due to the nonlinear I-V characteristics of these active electronic devices, antennas integrated with these semiconductor devices (especially varactors) produce signal distortion, which limits their application in high-power transmission [46,47].

Reconfigurable antennas based on LM provide a new technology that overcomes the above limitations. In LM-based reconfigurable antennas, the alterations in the length and position of the LM in a large amplitude greatly enhances the reconfigurable state of the devices. The realization of these reconfigurable states does not depend on nonlinear materials or mechanisms and these antennas also have higher linearity compared to antennas using semiconductor switches.

**Figure 10:**
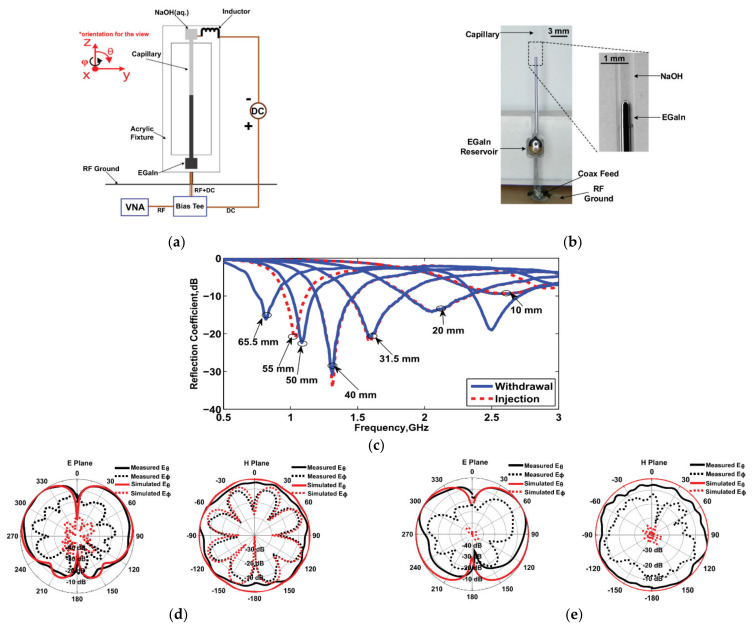
(**a**) Schematic of the tunable monopole antenna. (**b**) Photograph of the antenna, feed, and reservoir. (**c**) Reflection coefficient of different lengths. (**d**) Radiation patterns for 2.5 GHz with a length of 17 mm. (**e**) Radiation patterns for 0.96 GHz with a length of 55 mm. Reprinted with permission from Ref. [23]. Copyright 2015 AIP Publishing.

Using the electrochemically controlled capillarity (ECC) mechanism, bias voltage is used to control the electrochemical reaction on the surface of the LM immersed in the electrolyte solution. Under different DC bias conditions, the electrochemical reaction can remove or regenerate oxides on the surface of the LM, thereby enhancing or reducing the surface tension of the LM. The difference in surface pressure causes flow of the LM in the microfluidic channel. The process of using voltage to control the formation and removal of the oxide layer on the surface of the LM is reversible and repeatable, so the displacement of the LM in the microfluidic channel is reversible. Simultaneously, the LM can be pumped into and out of the microfluidic channel under electric control, and hence the LM can alter its position and length to a large extent. Electronically controlled microfluidic LM is capable of achieving continuous and large amounts of reconfigurable working conditions, which provides a new idea for the development of reconfigurable antennas and radio frequency devices. The ECC mechanism was used in [23] to achieve continuous reconfiguration of a monopole antenna, as shown in Figure 10. We used EGaIn to fill the liquid storage tank at the lower end of the capillary and connect to the SMA connector through the short copper wire in the liquid storage tank. The electrolyte filled the upper liquid storage tank to establish a DC current loop. When a positive DC potential was applied to the LM, we injected EGaIn into the glass capillary and replaced the electrolyte in the channel. EGaIn returned to the storage tank, reversing the polarity of the voltage. The measurement tuning range of the LM antenna was from 0.66 GHz to 3.4 GHz, and the cross-polarization gain was still greater than 10 dB.

**Figure 11:**
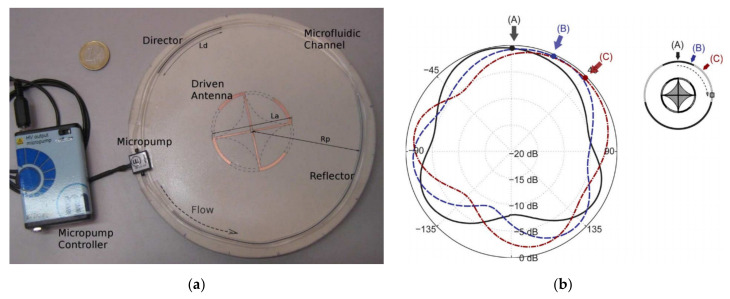
(**a**) Photograph of the microfluidic reconfigurable antenna. (**b**) Measured radiation patterns for configurations corresponding to rotations of 0°, 22.5° and 45°. Reprinted with permission from Ref. [50]. Copyright IEEE 2012.

A reconfigurable Yagi antenna based on LM in [48] consisted of an active dipole and a pair of stretchable passive parasitic dipoles. These dipoles were constructed with EGaIn embedded in the microfluidic channel. Both ends of the parasitic dipole were driven by two low-cost three-dimensional printing media rods. Rotating the rods at different angles resulted in different degrees of stretching on the stretchable dipole. A ~10 dB bandwidth was achieved in the 2.4–2.48 GHz WLAN band. The Yagi antenna in [49] was driven by a center-fed circular patch antenna and a c-shaped slot. By tuning the position of the LM alloy in the tube to adjust the polarization direction, the bandwidth could cover 4.3–5.3 GHz (21.2%). Ref. [50] proposed a new antenna reconstruction mechanism based on LM section displacement, as shown in Figure 11. The antenna was a new type of circular Yagi-Uda array, which was composed of a central drive element and a microfluidic channel composed of a movable LM parasitic part cyclically arranged around the central drive element. The movable parasitic indicator and reflector elements were realized by LM mercury (Hg), which guided the antenna beam through their variable positions. Antennas can realize the displacement of liquid elements more effectively than solid materials by applying precise microfluidic technology such as continuous flow pumping or electrowetting. Through the design, manufacture, and measurement of the reconfigurable radiation pattern antenna, the reconstruction mechanism was verified. The antenna operated at 1.8 GHz with a bandwidth of 4.0% and was capable of performing beam steering and fine-tuning within 360° ranges.

A LM-based multifunctional antenna that can achieve broadband frequency tuning, dual-band operation and polarization reconstruction, allowed impedance matching in a large frequency range [51]. Likewise, a planar LM parasitic antenna in [52] used the LM plug as a parasitic element instead of relying on the phase shifter for beam steering. The flow of LM in the channel guided the main beam on the E, H plane and the diagonal plane.

A polarized reconfigurable antenna that can achieve three polarizations of LHCP, RHCL and LP [53] had an aperture coupling composed of LM alloy and copper strip. The patch antenna, which was planned to reconfigure the position control of LM driven by pressure, had a center frequency of 2.45 GHz, and the radiation efficiency during measurement was higher than 90%. A microstrip circular patch antenna with polarization reconfiguration had a C-shaped groove in the center of the patch, using two putty containers and LM to switch between four different states [54]. Of the four states, when there was no LM in the container, LP was observed at 5.83 GHz; when two LM droplets were deposited in the container, CP was generated at 6 GHz; the RHCP was obtained when filling the rightmost container, and the LPCP was obtained when filling the leftmost container with LM. By changing the position of the LM on the top of the circular patch antenna with a C-shaped groove, the reconfigurability of frequency and polarization could be easily obtained.

**Figure 12:**
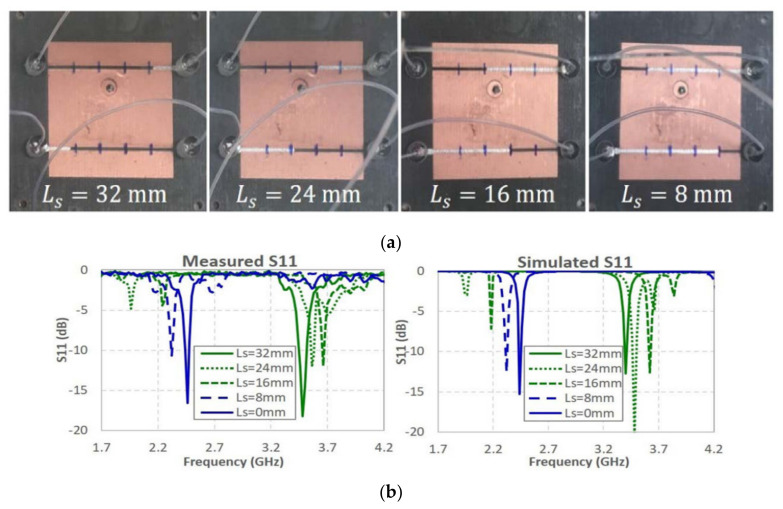
(**a**) Photograph of single-pair slot PASS with different Ls states. (**b**) Measured and simulated S_11_ for varied Ls. Reprinted with permission from Ref. [55]. Copyright IEEE 2019.

A wideband and continuous frequency reconfigurable patch antenna with switchable slots (PASS) was proposed in [55], as depicted in Figure 12. Frequency reconstruction relied on tuning and switching grooves achieved by continuously moving the volume of LM over channels covering narrow grooves. The patch antenna was designed with three pairs of composite slots, and the microfluidic channel was glued on the top of the metal layer. The physical reconstruction mechanism based on LM tuning provided the ability to continuously change the antenna characteristics in a very wide frequency range. Simulation and measurement results showed that by adjusting and switching the LM loading slot in the designed microfluidic channel, a wide frequency tuning bandwidth of about 70% and an instantaneous bandwidth of 2% were achieved without significant changes in the radiation pattern. Connecting the 3-D printed microfluidic module to the patch antenna on the PCB with a patterned adhesive layer, this manufacturing process was effective for constructing an EGaIn LM antenna with minimal skin residue. 

### 4.3. Microwave Devices

In recent years there has been growing interest in using LMs as tuning components for radio frequency/microwave applications such as filters [9,10,11,12], resonators [13] and frequency selective surfaces [14,15]. Compared with solid metal, LM not only has high conductivity but also has the natural advantages of good fluidity, controllable deformation and portability. When LM material is used in microwave devices and paired with elastic linings such as PDMS, the shape and size of the devices can be changed according to actual needs to meet the requirements of flexibility and multistate frequency band.

**Figure 13:**
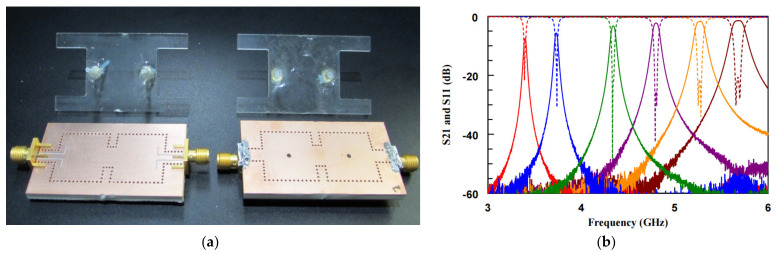
(**a**) Top (left) and bottom (right) views of the LM-based second-order bandpass filters. (**b**) Measured S-parameters of the proposed filter. Reprinted with permission from Ref. [9]. Copyright 2019 IEEE.

LM-based filters are usually combined with substrate integrated waveguide technology. Ref. [9] proposed a LM-based continuous tunable bandpass filter with a wide tuning range, as shown in Figure 13. The filter was realized by a square-type substrate integrated waveguide cavity resonator, and the resonator was capacitively loaded with a LM column in the center of the resonator. By changing the height of the LM column, thereby changing the loading capacitance, the resonant frequency of the resonator was continuously tuned. A second-order bandpass filter was implemented and measured. The filter was able to adjust the center frequency from 3.3 GHz to 5.8 GHz, confirming the wide-range tunability of the filter. This opened up a new direction for the LM tuning of wireless communication and radar systems that require frequency flexibility in the future.

Similarly, an LM-based (Galinstan) substrate integrated waveguide reconfigurable filter (BRF) consisted of four configurations [10], which were dual-band configuration (DBC), low-frequency configuration (LBC), high-frequency configuration (HBC), and no-band configuration (NBC). Reversible and repeatable switching between configurations was achieved by filling or emptying some of the LM in the through holes and channels, thereby adjusting the cavity boundary and the transmission line to obtain the desired response. For single-frequency and dual-frequency filter configurations, the insertion loss was less than 1.9 dB, and the return loss was greater than 16 dB when the center frequency was 4.6 and 7 GHz. For a non-band (or full stop) configuration, the isolation effect on the entire band between 4 and 8 GHz was better than 36 dB. This design provided a new filter implementation that can be used to alleviate the problems caused by overcrowding of the radio frequency spectrum in microwave wireless communications and multi-band radar applications. A LM tunable fourth-order integrated waveguide quasi-elliptic bandpass filter in [11] consisted of four frequency-tunable cross-coupled SIW resonators, creating multiple signal paths between input and output. The four SIW cavities had holes near their corners and were selectively filled with EGaIn to adjust the resonance frequency of the cavity. The measurement results showed that the filter could obtain four closely spaced passbands, and the center frequency could be discretely adjusted between 7.96 GHz, 8.12 GHz, 8.24 GHz and 8.39 GHz. A mechanically foldable and stretchable filter was proposed in [12], which had flexibility and mechanical stability, and its sensitivity to deformation was small. The filter injected LM into the PDMS microfluidic channel, which was implemented using soft lithography technology. Through simulation and experiments, the manufactured filter had an insertion loss of 1.3 dB at a center frequency of 2.1 GHz, and a return loss of more than 10 dB over 1.6–2.6 GHz. Under the maintained electrical specifications, it could be stretched up to 20% with a radius of 8.5 mm and a bend of 11.5 mm. This process had broad application prospects in emerging nonplanar applications such as wearable electronics, medical wireless sensors, and bioelectronics. The LM bandpass filter (BPF) in [31] had a similar structure to that in [12], but the center frequency was slightly shifted.

**Figure 14:**
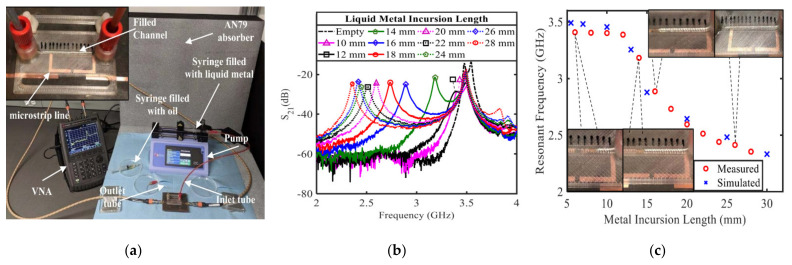
(**a**) Experimental device for measuring the S_21_ result of resonator proposed. Fabricated resonator filled with Galinstan and microchannels are shown in the upper left corner of illustration. (**b**) Resonator S_21_ spectra recorded by the VNA under different LM incursion length. (**c**) Record resonance frequencies of different metal lengths, including selected images. Reprinted with permission from Ref. [13]. Copyright 2020 IEEE.

Microwave split ring resonators (SRR) are limited in modern designs and cannot provide a fixed resonance response based on their size and material composition. Ref. [13] developed a frequency-tunable resonator structure that was frequency-tuned by integrating LM circuit components with continuously variable lengths. The microchannels were prepared by 3-D printing, filled with LM (Galinstan), and had a compact structure. In order to analyze the effectiveness of the tunable resonator structure, the S_21_ response was measured when the volume of LM injected into the channel was changed, and the trend between the length of the LM and the resonance frequency shift was given, as shown in Figure 14. Experimental analysis showed that the design could achieve continuous adjustment of the resonance frequency from 2.35 to 3.4 GHz (relative change was 45%), and the average sensitivity of the length between the resonator and the LM was 375 MHz/cm.

A frequency selective surface is a periodic array structure of one-dimensional or two-dimensional sub-wavelength metal elements on the surface of a medium. Ref. [14] proposed a physical flexible (bendable, foldable) gallium-based LM band stop frequency selective surface for PDMS package. LM was embedded in PDMS microfluidic channels replicated from deep-etched silicon molds. Using ultra-low temperature vacuum (5.75 × 10^−6^ Torr) at low temperature (10 K) to clean the packaged LM, a 5 × 5 FSS array was fabricated. The prepared FSS was characterized in a waveguide environment, and the simulated and measured S parameter values with stop FSS response were in good agreement. A flexible, adjustable, and tunable second-order bandpass FSS was proposed in [15], which used an elastomer microfluidic channel similar to [14] to encapsulate gallium-based LM. The method changed the position of the LM and adjusted the capacitor linearly, to achieve high-precision real-time continuous tuning.

**Figure 15:**
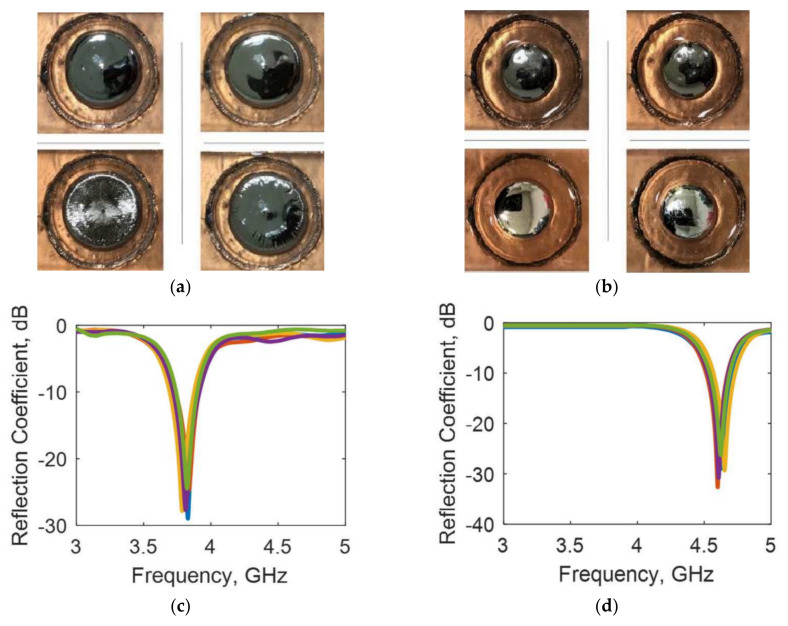
(**a**) EGaIn moved to a radius of 8 mm. (**b**) EGaIn moved to a radius of 6 mm. (**c**) Measured reflection coefficient of the unit cell with radius of 8 mm. (**d**) Measured reflection coefficient of the unit cell with radius of 6 mm. Reprinted with permission from Ref [27]. Copyright 2019 IEEE.

A two-dimensional tunable frequency selective absorber based on electrochemically driven LM was introduced in [27]. One unit of the absorber was composed of LM (EGaIn) droplets and sodium hydroxide solution, and the structure was composed of a circular chamber and small steps on a three-dimensional printing substrate. The radius of the LM could be controlled by electrochemical methods, as shown in Figure 15. The novelty of this design was its reconfigurable response, which depended on the size of EGaIn on the two-dimensional surface. By changing the radius of EGaIn, the cell of the frequency selective absorber could achieve four adjustable states in the simulation. The experimental results showed that the circular EGaIn patch could be easily generated and controlled by DC bias and produced an adjustable absorption peak of 25–30 dB.

### 4.4. Liquid Metal Sensors

A sensor is the detection unit of the devices to the external environment or the human body, and the measured information is obtained and converted into electrical signals or other forms of output signals to satisfy the transmission, storage, processing, display, and recording of information. Divided by use, sensors can be divided into force sensors, speed sensors, displacement sensors, humidity sensors, temperature sensors, pH value sensors, and ultraviolet sensors, among others. The most common LM sensors are strain sensors, which are important in studying changes of the human body in the medical field. In the human sensors can monitor parameters such as heart rate, facial expressions, and joint movements of fingers and knees. When measuring these physical parameters, people usually measure the frequency or magnitude of the stress or strain at the corresponding position, so stress-strain sensors are a very important category of LM sensors.

**Figure 16:**
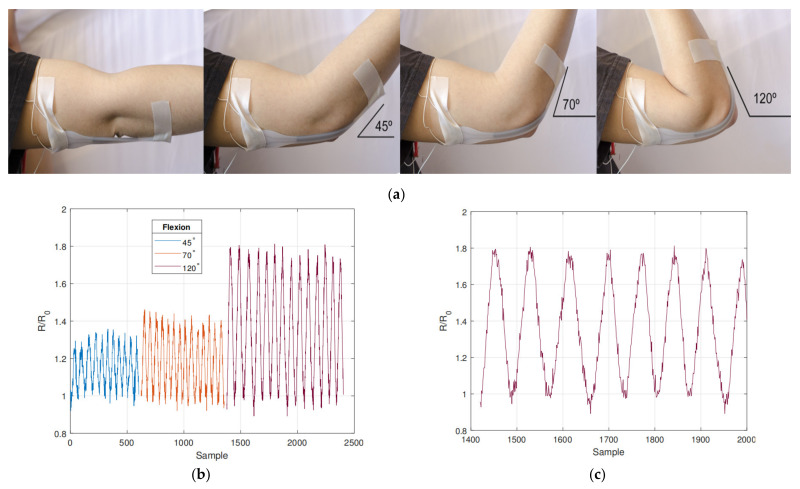
Resistive strain sensor attached to the elbow with three ranges of flexion. (**a**) Resistive strain sensor with elbow angle at 0, 45, 70, and 120 degrees. (**b**) Arm is moved from zero degrees to desired angle and back several times each, generating the waveform. (**c**) Data from repeated 120° flexion. Reprinted with permission from Ref. [26]. Copyright 2018 IEEE.

Stretchable electronic circuits and systems are essential for future wearable devices and smart textiles. Existing rigid and flexible manufacturing methods severely limit conformal deformation. A 3-D printing, highly stretchable strain sensor was proposed in [26]. The gallium-based LM paste for 3-D printing, in order to obtain better stability and rheological properties that were conducive to extrusion, added nickel nanoparticles and ultrasonic treatment. It was proven that the stable conductivity had close to zero hysteresis within 375 cycles at 200% strain. This device was used to measure the flexion angle of the elbow joint as shown in Figure 16, which provided proof of concept for the application of biomechanical sensors and wearable human-machine interfaces.

A stretchable capacitive strain sensor based on thermoplastic elastomer (TPE) microfluid embedded in LM was successfully prepared [16]. Due to the unique self-adhesive properties of the TPE material, the capacitance of the sensor was increased by increasing the number of TPE layers. The change of sensor capacitance was linear with the strain and with small hysteresis. The measurement factor of the sensor was close to the theoretical maximum of 1, and the capacitance of the sensor was very stable and repeatable after being stretched and released for 5000 cycles. A soft micro-finger was integrated by a pneumatic balloon actuator (PBA) and a strain sensor using LM [17]. Both the actuator and the sensor consisted of simple structures, with a cavity or channel sandwiched between two thin films. By injecting LM into the microchannels in batches, LM-based strain sensors were prepared. The LM-based strain sensor (50 μm × 50 μm × 2 mm) could successfully detect the movement of PBA (560 μm × 130 μm × 900 μm). The count factor of the strain sensor was estimated to be 0.8, and the sensors and wiring were excellent candidates for soft robotics applications. To quantify the strain naturally occurring in wearable electronic devices, such as the strain of the human body or the body of a robot, strain sensors with the same or greater physical characteristics are required. In [56] is reported a new type of micro-etching preparation method using soft lithography technology to prepare a super-stretchable strain sensor, which used Ecoflex silicone encapsulation pattern LM. Compared with the prior art, the sensor can be stretched 300% and twisted 180°, with repeatability error of the stretch release process less than 3% and a sensitivity parameter measurement coefficient up to 8. The design performance was different from PDMS-based sensors, carbon nanotubes and graphene sensors, showing extreme mechanical properties and high sensitivity even under multiple cycles of 100% strain load.

**Figure 17:**
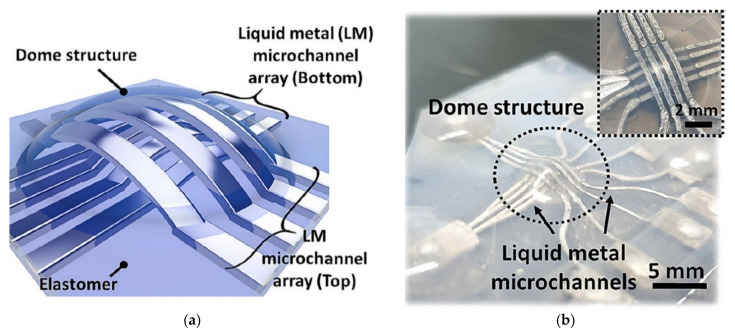
LM-based multiaxial force sensor with three-dimensional dome structure: (**a**) Schematic of the proposed sensor; (**b**) photograph of the proposed sensor. Reprinted with permission from Ref. [59]. Copyright 2021 Springer Nature.

In [57], the sensitivity of a highly scalable LM (Galinstan) sensor for temperature and humidity was studied, and it was found that the resistance of the sensor had a quasi-linear relationship with temperature changes without any hysteresis. The parallel design had a higher sensitivity to wetting, which was believed to be due to the specific path of the electric field lines. It was observed that as the sensor area was wetted by tap water, the capacitance changed as high as 76%. When a compressive force was applied, the capacitance values of all sensors were nonlinearly dependent and had small hysteresis. In capacitive sensing mode, all sensors were particularly sensitive to small forces (<4 N). The design can be used for the development of electronic skin and artificial nervous systems for biomedical and soft robotics applications. A new type of flexible pressure sensor based on PDMS and EGAIn was developed to detect various application pressures [58]. The LM-based electrodes were designed to form four capacitors. By studying the capacitance response of the device under different applied pressures, it was proved that the average capacitance change of the manufactured pressure sensor was linear, the sensitivity was 0.11%/MPa, and the correlation coefficient was 0.9975. The results obtained proved the feasibility of using LM-based electrodes to fabricate flexible pressure sensing devices. To be able to accurately monitor the external environment, a full-soft multiaxis force sensor based on a LM microchannel array consisted of a stretchable elastomer and Galinstan, as shown in Figure 17 [59]. The LM microchannel array was fabricated in multiple layers and positioned along the three-dimensional dome structure to detect multidirectional forces. By adjusting the height of the dome, the response of the multi-axis sensor to advection can be controlled. The sensor had high mechanical friction and electromechanical durability and can be applied in various research, including soft robots, wearable devices, and smart prostheses for artificial skin applications.

**Figure 18:**
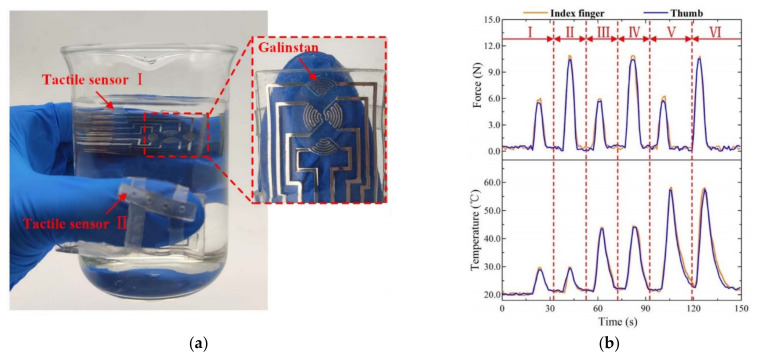
(**a**) Tactile sensors worn on the index finger and thumb of the human hand that were used to grasp a beaker of water; (**b**) Results of sensing force and temperature. Reprinted with permission from Ref. [60]. Copyright 2021 IEEE.

Wearable tactile sensors can be used for tactile perception and are widely used in applications such as soft robots and smart prostheses. A wearable tactile sensor based on LM (Galinstan) can sense temperature and contact force simultaneously, as shown in Figure 18 [60]. LM was injected into the fingerprint-shaped microfluidic channel, and the output voltage signal of temperature and contact force sensing was decoupled through the structural design of a Wheatstone bridge circuit, fingerprint pattern microfluidic channel and top elliptical protrusion. The tactile sensor had a high force sensing sensitivity of 0.32 N^−1^, and the temperature sensing sensitivity was 0.41% °C^−1^ at 20 ∼ 50 °C, and 0.21% °C^−1^ at 50 ∼ 80 °C. The two wearable tactile sensors were only 1.2 mm thick and were worn on human index fingers and thumbs to detect temperature changes and contact forces during gripping applications. Experimental results showed that the developed LM-based tactile sensor had high flexibility and durability in more than 200 cycles of loading tests. Therefore, the developed wearable tactile sensor has great potential in robot operation and human medical condition perception.

### 4.5. Wearable Devices and Flexible Circuits

Soft wearable electronic material products are developing rapidly. The resurgence of interest in LMs in the sensing and wiring functional properties of soft wearable electronic materials has brought tremendous advances to wearable electronics and materials. Various forms of LM enable many routes to manufacture flexible sensors, circuits and wearable functional wearable devices with many ideal properties [61].

**Figure 19:**
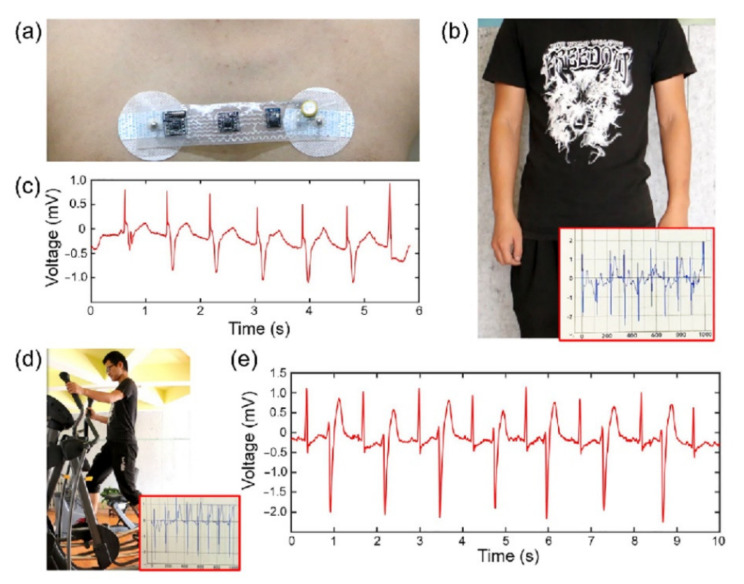
(**a**) LM e-Skin ECG device. (**b**) When the human body is stationary, the LM e-Skin collected the signal and displayed it on the computer. (**c**) ECG data processed by MATLAB. (**d**) When the human body moved, the LM e-Skin collected signal and displayed it on the computer. (**e**) ECG data in motion processed by MATLAB. Reprinted with permission from Ref. [64]. Copyright 2018 Springer Nature.

The implementation and exploration of LM in soft electronic products, especially electronic skin is rapidly increasing. Due to increasing research on gallium-based alloys, this field has received special attention. Compared with the toxicity of mercury, gallium-based alloys are much safer. The LM alloy of gallium provides unique physical and chemical properties for electronic skin. These properties stem from their high thermal conductivity and electrical conductivity, and the fact that LMs are an electronic melt compared to ionic liquids. LM, whether in volume or particulate form, provides good stretchability and allows the formation of a plastic electronic skin. Therefore, it provides an opportunity for the development of components with extraordinary flexibility, plasticity and skin compatibility [62]. Electronic skin has a wide range of applications in biomedical equipment, smart robots and wearable devices, and has mechanical properties similar to human tissues. Ref. [63] developed a rapid preparation method for flexible electronic skin based on LM, which can be used to produce flexible patches with good electrical stability and radiological imaging capabilities for detecting body surface temperature. In addition, a circular LM flexible patch was produced in [63], which can be applied to the surface of a substance or the human body. Since the patch has a strong barrier to X-rays, it can achieve accurate positioning of lesions in the body with the help of CT. The LM used in the method was a semi-LM material (Cu-EGaIn) with high viscosity and flexibility prepared by mixing gallium-based alloy and solid metal particles (Cu) at room temperature. In this research, this material was printed on the surface of a flexible substrate through a toner mask roll coating to make flexible wearable electronic devices. Traditional rigid electronic products are usually inconsistent with soft skin and are often unable to carry out accurate physiological monitoring and precise treatment. Electronic skins made of conductive and stretchable materials provide mechanical compliance for the manufacture of flexible and compatible wearable devices. In [64], a highly conductive and stretchable wearable LM electronic skin was demonstrated, which can be used for physiological signal monitoring. The LM circuit (LMC) was embedded in a silicone rubber film, and the serpentine structure design allowed the LMC to maintain good conductivity and function under a strain of more than 100%. Simultaneously, a wearable electrocardiogram (ECG) recording device was produced and tested, as shown in Figure 19. The device could obtain a stable signal during the real-time measurement of physical activity, and it had excellent flexibility and stability. In the future, this LMC-based flexible electronic skin can be widely used in wearable human body networks, with sensing, monitoring and treatment functions.

**Figure 20:**
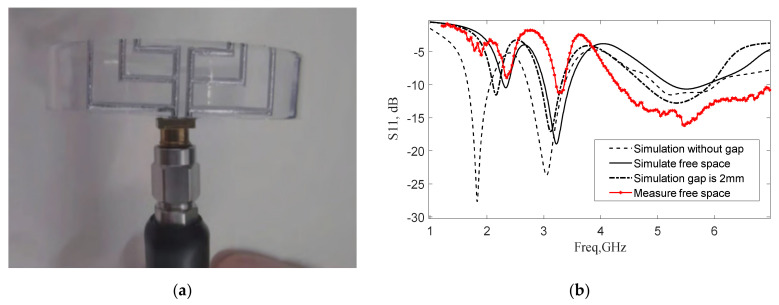
(**a**) 3-D printed tri-band LM antenna. (**b**) Reflection coefficients of the tri-band bracelet antenna. Reprinted from Ref. [65].

We proposed a 3-D printed tri-band LM antenna for a wrist-worn sensor that worked at 2.4, 3.3 and 5.5 GHz, as shown in Figure 20 [65]. The dielectric substrate and the micro-passband cavity that conformed to the human wrist were processed by 3-D printing technology, and then the micro-passband cavity was quickly filled with LM to form the radiating conductor of the antenna. The influence of the thickness of the 3-D printing substrate on the antenna radiation efficiency, and the interaction between the antenna radiation pattern and human tissues, were studied. When there was no gap between the human tissue and the bracelet, the medium loading effect of the human tissue was the largest, and the antenna resonance frequency shifted the largest. When this distance gradually increased to 2 mm, the antenna resonance frequency became smaller. Therefore, the influence of human tissue on the resonant frequency of the antenna was related to the gap distance between the human tissue and the bracelet. This verified the application of antennas based on 3-D printing technology and LM in the field of wearable and conformal electronic components. An antenna with more flexible structure and performance can be made according to actual application scenarios.

The technology in [66] used an LM mask printing method, which allows the rapid manufacturing of flexible electronic devices on the fabric and had good stretchability. Offset printing polymethacrylate (PMA) on the fabric was used to improve the adhesion of LM (EGaIn) to the fabric. Combined with surface mold printing, LM can be directly and quickly printed on fabrics with PMA glue to manufacture flexible electronic devices, providing a valuable prospective method for personalized wearable medical equipment. In addition, the method has broad application prospects in the mass production of smart electronic fabrics. With the injection of LM microchannels to form wires, Ref. [9] proposed a method of connecting hundreds of soft and scalable electronic components of micro-scale electrical components through selective batching of LM interconnections. This method did not form independent interconnections in series, such as 3-D printing or traditional microfluidics but disconnected the short-circuited LM interconnections in parallel to form a circuit, and could form ~300 isolated electrical connections. The success of this method opens up a new path for the large-scale and high-density LM interconnection of complex scalable electronic circuits and robotics technology. When equipped with large-scale and high-density robust LM interconnects, it stimulates the development of soft and scalable electronic circuits and robots to higher complexity. With the rapid development of wearable electronic devices, wearable fuel cells as smart energy sources are receiving more and more attention due to their high energy conversion efficiency, moderate operating temperature, and easy handling [67]. To solve the limiting factor of rigid electrodes of fuel cells, eutectic gallium indium LM was used for high-performance wearable and rechargeable fuel cells. Because the material had excellent deformability and redox ability, the power density of the fuel cell was as high as 72.8 mWcm^−2^, and it could be used as a rechargeable metal-air battery under 2 mAcm^−2^ to circulate stably for 96 h. This new type of renewable LM anode, based on high-efficiency wearable liquid fuel cells, has broad prospects as a shape-variable energy supply for bionic soft robots and wearable devices.

A liquid-state light sensor and an optical memory which was turned on and off by ultraviolet and blue light irradiation were discussed in [68]. Ionic liquid 1-butyl-3-(4-phenylazobenzyl) imidazolium bis (trifluoromethanesulfonyl) amide ([Azo][NTf2]) was used to realize the function of light sensor and optical memory. This ionic liquid was light-responsive, and could be controlled by ultraviolet or blue light irradiation to control reversible isomerization. In addition, it utilized a liquid heterojunction that sensed the interconnection between the ionic liquid and the LM. The liquid heterojunction in the microchannel was essential to prevent the mixing of the two liquid components, especially when the completed device underwent mechanical deformation. Two important technologies (photo-switchable ionic liquids and heterojunctions) realized liquid-state optoelectronics based on liquid materials.

**Figure 21:**
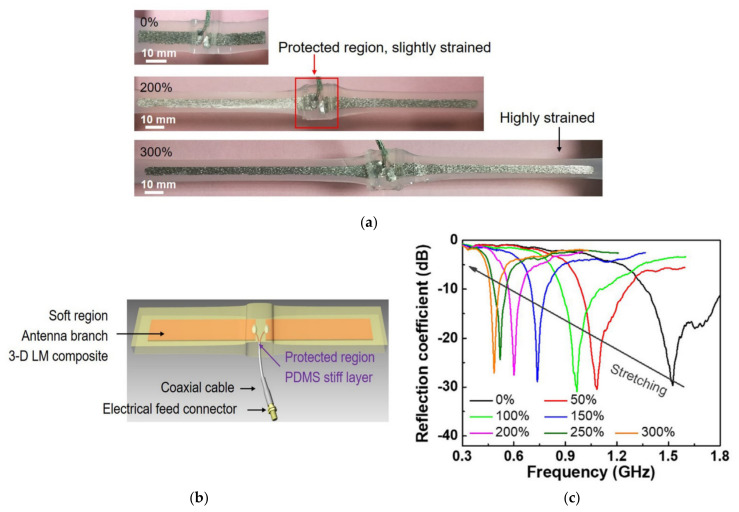
(**a**) Antenna under different strains. (**b**) Schematic diagram of the dipole antenna. (**c**) Frequency response of the reflection coefficient of the antennas under different tensile strains. Reprinted with permission from Ref. [69]. Copyright 2020 Elsevier.

A tunable dipole flexible antenna that can be stretched up to 300% was developed in [69], as shown in Figure 21. A 3-D LM network was embedded in an elastically soft elastomer as a conductive branch, and a rigid elastomer was coated on the rigid feed connector area as a protective layer to achieve mechanical fatigue resistance and a constant reflection coefficient. The antenna simultaneously presented a high-quality reflection coefficient around 30 dB and a wide adjustable resonant frequency from 1.55 to 0.45 GHz. This design is very promising for flexible electronics and wireless strain sensors.

## 5. Discussion and Conclusions

This paper reviews different aspects of LMs, including material properties, fabrication, and functionalities. Common LMs, including Galinstan and EGaIn, due to their high thermal conductivity, high electrical conductivity, low viscosity and high fluidity, are becoming an ideal candidates in a wide range of fields.

The fabrication technology of LM includes 3-D printing [25,26,27,28], soft lithography [31,32] and spray printing [4,5,6]. To manufacture complex three-dimensional antennas, a cavity can be made by 3-D printing, and the pressure difference used to push the metal into the cavity [25]. For the 3-D printed gallium-based LM paste, to obtain better stability and rheological properties conducive to extrusion, nickel nanoparticles and ultrasonic treatment were added [26]. In the EGaIn thin line pattern production method based on soft lithography technology, the size of the produced thin lines are scalable, uniform and without residue, and the line width on the same soft substrate is from a single micron to a few millimeters [3]. Spray printing can be directly applied to various substrates, and LM droplets can be sprayed on the cloth substrate [4], sprayed and wiped on the PDMS substrate [5], or quantitatively sprayed and packaged in silicone elastomer in combination with silicone [6].

Due to its natural advantages of high electrical conductivity, high thermal conductivity, fluidity, and controllable deformation, LM has been widely used in switches [39,40,41,42,43], reconfigurable antennas [48,49,50,51,52,53,54,55], microwave devices [10,11,12,13], sensors [57,58,59,60], and wearable devices and flexible circuits [62,63,64]. Gallium-based LM can be used as a substrate integrated waveguide switch [39] and can also be used as a thermal switch controlling heat transfer spatially and temporally [42]. The LM switch combined a dielectric spacer on the radio frequency path with a capillary groove filled with electrolyte. The introduction of the dielectric spacer can reduce insertion loss and allow low-power electric drive [41]. When the LM switch was applied to the spoof surface plasmon transmission line, the open state was obtained through the flow of mineral oil and LM [43]. LM switches can avoid electrical contact gaps between traditional semiconductor switches, which can significantly reduce back radiation [44]. In LM-based antennas, the alterations in the length and position of the LM in a larger amplitude greatly enhanced the reconfigurable state of the devices with a larger frequency adjustment range. The realization of these reconfigurable states did not depend on nonlinear materials or mechanisms, so these antennas also had higher linearity than antennas that used semiconductor switches. A monopole antenna using the ECC mechanism allowed continuous reconfiguration of the frequency band and radiation pattern [23]. The position of the LM was changed to adjust the polarization direction, and the Yagi antenna could cover the bandwidth of 4.3–5.3 GHz [49]. The movable parasitic indicator and reflector elements in the new circular Yagi-Uda array were realized by LM mercury (Hg), which guided the antenna beam through their variable positions, and could perform beam steering and fine-tuning within 360° [50]. The study [52] proposed a planar LM parasitic antenna. Instead of relying on the phase shifter for beam steering, it used the LM plug as a parasitic element. The flow of LM in the channel guided the main beam on the E, H plane and the diagonal plane. In [63], a circular LM flexible patch was developed, which can be applied to the surface of a substance or human body. Because the patch had a strong barrier to X-rays, accurate positioning of the lesions in the body can be achieved with the help of CT. The LM used in the method was a semi-LM material (Cu-EGaIn) with high viscosity and flexibility prepared by mixing gallium-based alloy and solid metal particles (Cu) at room temperature. The LM mask printing method allows the rapid manufacturing of flexible electronic devices on the fabric and had good stretchability [66]. The strain sensors proposed in [16,26] had good mechanical stability and a high measurement factor. A soft multi-axis force sensor based on an LM microchannel array could detect multi-directional force [59]. A wearable tactile sensor based on LM (Galinstan) had high flexibility and stretchability, and could sense temperature and contact force simultaneously [60]. Filters based on LM are usually combined with substrate integrated waveguide technology, such as the bandpass filters proposed in [9,10]. In [13], a frequency-tunable resonator structure was developed, and the frequency was tuned by integrating LM circuit components with continuously variable length. Ref. [27] introduced a LM-based two-dimensional tunable frequency selective absorber based on electrochemical driving. The reconfigurable response depended on the size of the LM (EGaIn), and the radius of the LM could be controlled by electrochemical methods.

Electromagnetic wave propagation technology based on metamaterials had narrow operating bands, which could be adjusted by introducing LM [70]. LM-based flexible electronics and metamaterials were combined, mechanical deformation was coupled with LM microchannels, and electrical conduction was controlled by shrinking and releasing the LM embedded in the soft material [71]. The introduction of LM into the soft polymer structure could achieve a significant toughening effect. The new LM filled polymer microlattice metamaterial had mechanical recoverability and a shape memory effect adjusted by heating [72]. High-efficiency tunable electromagnetic absorption metamaterials utilized the extraordinary fluidity and high conductivity of LMs to achieve good broadband tunable microwave absorption [73].

In short, due to the various excellent properties of LM: fluidity at room temperature, high conductivity, nontoxicity, and stable chemical properties, functionalities and applications based on LM are spreading across many fields including cognitive radio and spectrum aggregation wireless systems, indoor rooftop antenna network applications, biomedical equipment, smart robots and wearable devices. Currently LM-based electronics are mostly conceptualized demonstrations of novel structures or functionalities, while practical applications of such devices still face challenges. Compared with rigid or engineering substrates, flexible electronics using polymers or 3-D printed arrangements have lower mass density and lack long-term air tightness to oxygen, which potentially raises concerns of long-term durability. Durability of such devices can be improved by adding chemical composites or enhancing the micro-structure of materials, which requires corporate research in chemical and electrical engineering. With further understanding and research on LM, and gradually overcoming technical difficulties, it is believed that LM will have very broad uses in the future, especially for applications requiring flexible electronic devices and continuously tunable devices.

## Figures and Tables

**Table 1:** Physical properties of commonly used liquid metals. Reprinted with permission from Ref. [20]. Copyright 2018 John Wiley and Sons.

	Hg	Ga	EGaIn	Galinstan
Melting point [°C] [20]	−38.8	29.8	15.5	−19
Boiling point [°C] [20]	357	2204	2000	1300
Viscosity [10^−7^ m^2^ s^−1^] [20]	13.5	3.24	2.7	2.98
Surface tension [N m^−1^] [20]	0.5	0.72	0.624	0.533
Electrical conductivity [10^6^ S m^−1^] [20]	1.0	3.7	3.4	3.1
Thermal conductivity [W m K^−1^] [20]	8.34	29.4	42.2	44.8

## Data Availability

Not applicable.
